# Glucose uptake is essential for *Brucella abortus* growth in the extracellular space of the murine placenta

**DOI:** 10.1128/iai.00060-25

**Published:** 2025-03-12

**Authors:** Leticia Lázaro-Antón, Thaynara Parente de Carvalho, Eric Pirillo, Mariana X. Byndloss, Vladimir E. Diaz-Ochoa, Briana M. Young, Renato de Lima Santos, Renée M. Tsolis

**Affiliations:** 1 Department of Medical Microbiology and Immunology, School of Medicine, University of California 272916 , Davis, California, USA; 2 Departamento de Clínica e Cirurgia Veterinárias, Escola de Veterinária, Universidade Federal de Minas Gerais 154001 , Belo Horizonte, State of Minas Gerais, Brazil; 3 Department of Pathology, Microbiology and Immunology, Vanderbilt University Medical Center 204907 , Nashville, Tennessee, USA; 4 Howard Hughes Medical Institute, Vanderbilt University Medical Center 12328 , Nashville, Tennessee, USA; 5 Department of Pathology, Microbiology and Immunology, School of Veterinary Medicine, University of California 240470 , Davis, California, USA; Stanford University School of Medicine, Stanford, California, USA

**Keywords:** placental immunology, *Brucella*, glucose, metabolism, placental infection, trophoblast

## Abstract

*Brucella abortus* infects the placenta of its natural bovine host, which results in abortion and transmission of infection to other cattle and to humans. While the metabolism of *B. abortus* during chronic infection of the mononuclear phagocyte system has been studied, the nutrients fueling growth of *B. abortus* in the placenta are unknown. We found that in mice, glucose is an important carbon source for *B. abortus* in the placenta. A *gluP* mutant lacking a major facilitator superfamily protein required for glucose uptake had diminished growth in the placenta of pregnant mice and caused reduced inflammatory pathology and fetal demise. The *gluP* mutant was able to replicate intracellularly in a trophoblast cellular model and to cause trophoblast cell death in infected placentas. Attenuated growth of the *gluP* mutant was maintained in mice conditionally deficient for peroxisome proliferator-activated receptor γ in macrophages, suggesting that M2-like macrophages were not the major site for glucose-dependent growth of *B. abortus* in the placenta. Our results show that the infected placenta contains multiple distinct nutrient niches and that glucose utilization within the interstitial space of the placenta is an important process contributing to bacterial growth and fetal demise during placental *B. abortus* infection.

## INTRODUCTION

Maternal health status during pregnancy is crucial to the development of the placenta and fetus and a successful pregnancy outcome. During pregnancy, the placenta can become infected by bacteria, parasites, or viruses that elicit pathological responses, including abortion, perinatal infant mortality, malformations, or transmission of the pathogen to the newborn (1). Placental colonization is a feature of infections by viruses such as Zika and cytomegalovirus, pathogenic protozoa such as *Toxoplasma gondii*, as well as bacteria, including *Coxiella burnetii*, *Listeria monocytogenes*, and *Brucella* spp. (1, 2). While the tissue organization of the placenta at the maternal-fetal interface differs between humans, ruminants, and mice, a shared feature of several of these infections across host species is replication within populations of placental cells that include macrophages and trophoblasts (3).

*Brucella abortus* infects the placenta of its natural bovine host, where it replicates within a population of trophoblasts, triggering necrotic cell death and inflammation. This host response to infection is thought to disrupt the fetal-maternal interface that supports fetal growth, resulting in abortion (4, 5). If inflammatory pathology is less severe, calves may be born weak or may die after birth, and viable calves may be born infected with *B. abortus* (4, 5). Infected or aborted calves can transmit *B. abortus* infection to humans through occupational contact with infected tissues. The infection of pregnant women with *Brucella* spp. is understudied. However, a few studies have reported adverse pregnancy outcomes associated with infection by *Brucella* spp., including miscarriage, preterm delivery, and vertical transmission to the fetus (6, 7).

While the immunologic basis for *B. abortus*-induced inflammation in the placenta has been studied using a mouse infection model (8–11), our understanding of the metabolic requirements for bacterial growth in this organ is very limited. Pregnant mice share several features of the natural bovine host for *B. abortus*, including placental tropism and extensive multiplication of *B. abortus* in trophoblasts, with bacterial numbers in the placenta far exceeding those in the spleen (8, 12). This intense colonization and replication within trophoblasts lead to necrotizing inflammation and subsequent cell death, inducing placental inflammation—responses that promote fetal loss and transmission in cattle, the zoonotic reservoir host (9, 13, 14).

Studies of metabolism and localization of *B. abortus* during systemic infection have provided important insights into nutrients available for bacterial growth in the mononuclear phagocyte system (13, 15–18). In contrast, the environment of the placenta is very different, as it is a temporary organ formed at the maternal-fetal interface by both fetal and maternal cells and is rich in hormones and nutrients that support fetal development. Little is known about the metabolic basis by which *B. abortus* exploits this environment to support the rapid bacterial growth that is crucial for its transmission both within the zoonotic reservoir and to humans. Erythritol has been proposed as an important carbon source in the ruminant placenta as it was found to be abundant in bovine fetal cotyledons, a site of extensive *B. abortus* growth, and is the preferred carbon source for *B. abortus* growth *in vitro* (19, 20). In this study, we aimed to understand the metabolic requirements of *B. abortus* infecting the murine placenta and to shed light on the interplay between the bacterium and the maternal-fetal interface.

## RESULTS

### Glucose uptake drives *B. abortus* growth in the extracellular space of the placenta and contributes to fetal loss

**Fig 1:**
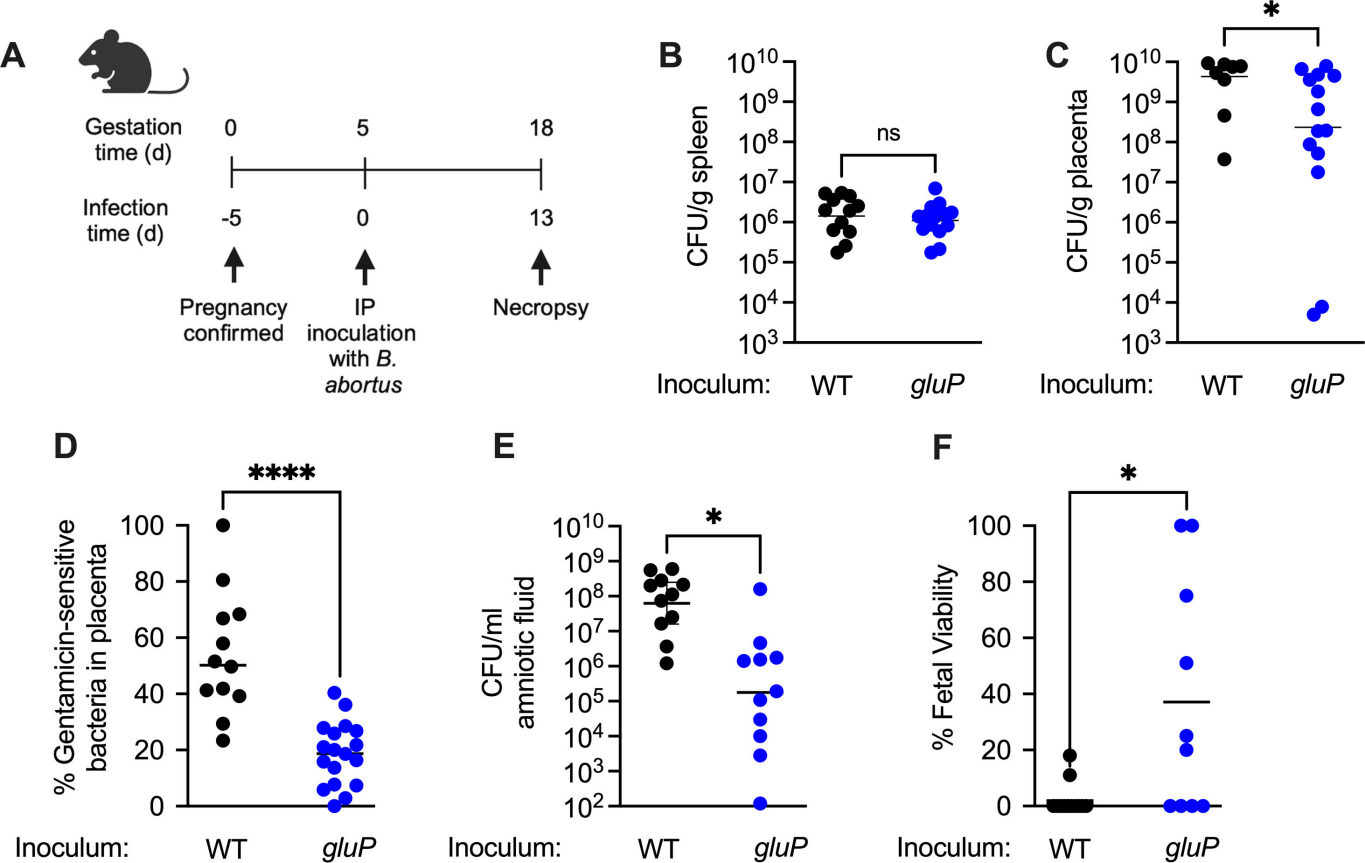
Glucose transport contributes to the growth of *B. abortus* in the extracellular space of the placenta and to fetal loss in *B. abortus-*infected pregnant mice. (A) Schematic representation of the experiment. Pregnant C57BL/6J mice were inoculated at 5 days of gestation via the i.p. route with either *B. abortus* 2308 WT or isogenic *gluP* mutant, and placentas were sampled at 18 days post-infection for analysis (made with BioRender). (**B and C**) Recovery of *B. abortus* 2308 WT and *gluP* mutant from spleens (**B**) and placentas (**C**) of mice infected with *B. abortus* WT or isogenic *gluP* mutant (*n*  =  12 for the WT-infected and *n*  = 16 for the *gluP* mutant-infected group). *P < 0.05; ****, *P* < 0.0001. (D) Proportion of extracellular (gentamicin-sensitive) *B. abortus* in placentas from infected mice (*n*  =  12 for the WT-infected group and *n*  = 16 for the *gluP* mutant-infected group). *P < 0.05. Significance of differences was analyzed using a Mann-Whitney test. (**E**) Recovery of *B. abortus* WT and *gluP* mutant from amniotic fluid after 18 days of gestation. *P < 0.05 (*n* = 11). (**F**) Fetal viability at 18 days of gestation in pregnant mice infected with *B. abortus* WT or *gluP* mutant (*n* = 11). *P < 0.05 using one-way analysis of variance. Values (black and blue circles) represent individual mice and means (black bars).

We previously demonstrated that during chronic infection in mice, survival of *B. abortus* within phagocytes at systemic sites depends on intracellular glucose availability (21). However, in the placenta, studies of carbon utilization by *B. abortus* have focused primarily on erythritol (13, 18, 20, 22), and it is not known if utilization of other carbon sources plays a role in establishing placental infection. To determine whether glucose utilization could promote growth of *B. abortus* in the placenta, we inoculated pregnant mice at 5 days of gestation via the intraperitoneal (i.p.) route with either the *B. abortus* wild-type (WT) strain 2308 or an isogenic mutant defective for the major facilitator superfamily transporter GluP (*gluP* mutant), and collected splenic or placental tissues at 18 days of pregnancy (Fig. 1A). The sites in the materno-fetal unit at which we assessed *B. abortus* colonization are shown in Fig. S1. Consistent with our previous results from acute infection of non-pregnant mice (21), the *gluP* mutant colonized the spleen at levels similar to WT (Fig. 1B). In contrast, a marked reduction in placental colonization of the *B. abortus gluP* mutant compared to WT was observed (Fig. 1C). These results showed that in pregnant mice, glucose uptake is required for growth of *B. abortus* in the placenta but not in the spleen.

*Brucella* is known to be associated with an extracellular or interstitial niche during placental infection of both mice and ruminants (reviewed in reference 14). To determine whether the glucose uptake defect of the *gluP* mutant affected *B. abortus* growth specifically within cells or in the interstitial space location, we treated placentas collected at necropsy with gentamicin, which preferentially kills extracellular *B. abortus*, and enumerated CFU of *B. abortus* in untreated tissue, to determine the total bacterial load and in gentamicin-treated tissue, to determine the number of intracellular (gentamicin-resistant) bacteria. These values were used to approximate the percentage of bacteria ([total CFU-intracellular CFU] / total CFU) in the tissue homogenate that are resistant to gentamicin killing and therefore likely to be extracellular. Compared to WT *B. abortus*, the *gluP* mutant in placental tissue homogenates was protected to a greater extent from killing by gentamicin, suggesting that more of the bacteria were within placental cells (Fig. 1D). We also assayed recovery of *B. abortus* from the amniotic fluid, collected from the space between the amniotic sac and the pup (Fig. S1), and consistent with results from the placenta, the *gluP* mutant also exhibited a significantly reduced bacterial load in the amniotic fluid (Fig. 1E). Taken together, these results suggest that glucose uptake fuels growth of *B. abortus* in the extracellular or interstitial space of these sites.

In pregnant cows, a consequence of the massive replication of *B. abortus* in the placenta is abortion of the calf, which transmits the infection to other susceptible hosts, including bovines and humans. To investigate the role of glucose uptake in contributing to fetal death caused by *B. abortus*, we measured the viability of pups from dams infected with wild type or *gluP* mutant, based on the presence of fetal movement, heartbeat, fetal size, and skin color, as previously described (9). While pups from dams inoculated with wild-type *B. abortus* were mostly non-viable, pups from dams inoculated with the *gluP* mutant had a significantly higher survival rate in the majority of the mice (Fig. 1F). Together, these data demonstrate that *B. abortus* requires glucose transport to establish infection in the placenta and that the growth of *B. abortus* to the high levels recovered from the placenta of mice (Fig. 1C) depends on acquiring glucose in an extracellular or interstitial replication niche. Furthermore, these results indicate that the development of the characteristic abortifacient phenotype of *B. abortus* is dependent on glucose transport.

### Neither proliferator-activated receptor γ expressing macrophages nor trophoblasts constitute the predominant niche within the placenta for glucose-dependent growth of *B. abortus*

Within infected placental tissue, *B. abortus* can be found in association with trophoblasts, a specialized cell that develops only during pregnancy, and within phagocytes (reviewed in reference 14). Therefore, we investigated whether *B. abortus* required glucose for replication in one of these intracellular niches.

**Fig 2:**
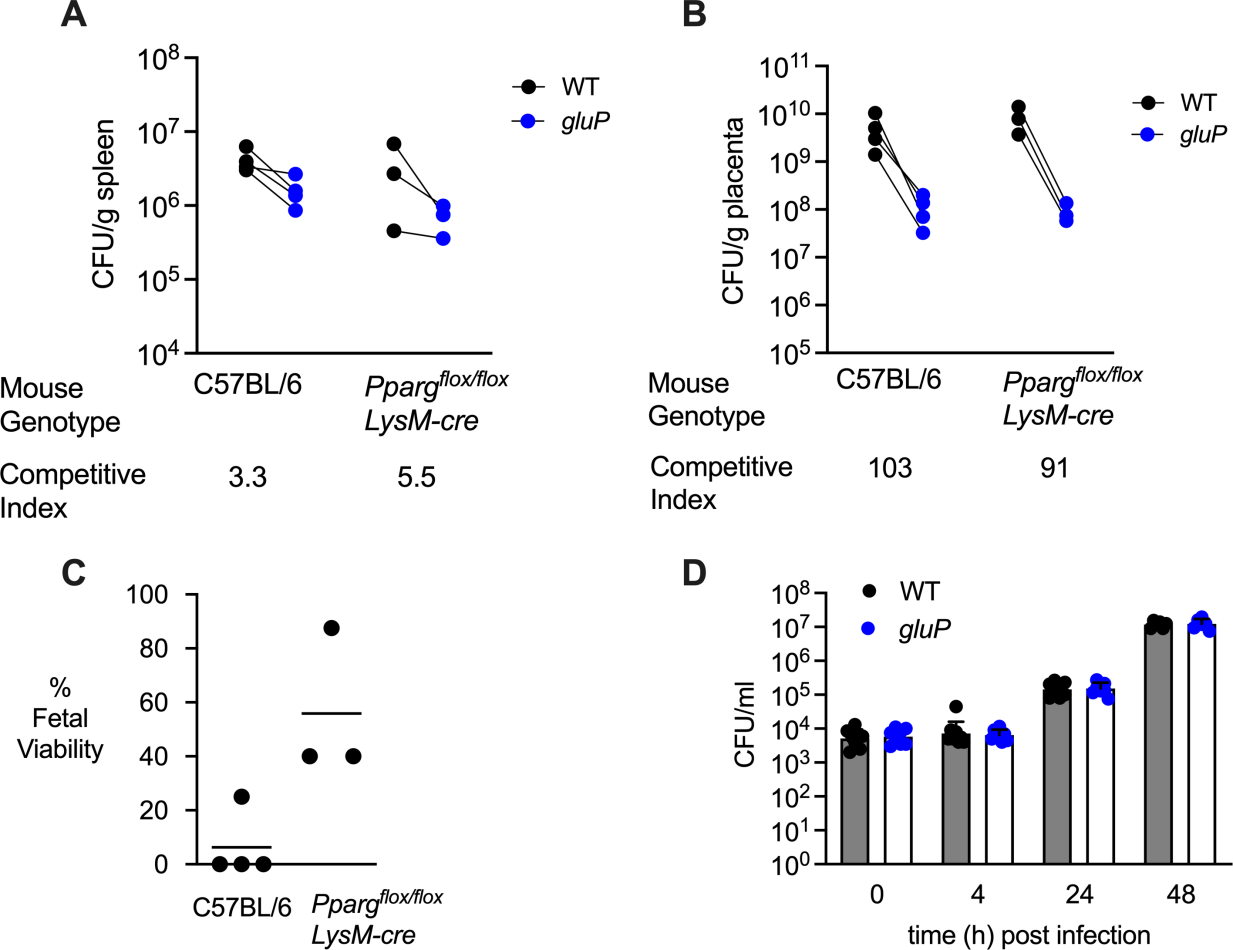
Neither PPAR-γ-expressing alternatively activated macrophages nor trophoblasts constitute the primary niche within the placenta, where *B. abortus* relies on glucose for its growth. (**A and B**) Competitive index (ratio of WT to *gluP* mutant) in spleens (**A**) and placentas (**B**) of C57BL/6J WT mice (*n* = 4) and *Pparg*^fl/fl^ LysMcre+/- mice (*n* = 3) infected with a 1:1 mixture of *B. abortus* 2308 WT and *gluP* mutant at 5 days of pregnancy and euthanized at 18 days of gestation. (C) Viability of fetuses recovered from C57BL/6J WT mice (*n* = 4) and *Pparg*^fl/fl^ LysMcre^±^ mice (*n* = 3) in the same experiment. (D) Intracellular CFU of wild-type *B. abortu*s 2308 WT and *gluP* mutant in infected Bewo cells over time. Results shown are combined from three independent experiments and are displayed as geometric mean ± SD.

To determine whether M2-like macrophages are the predominant glucose-containing niche for *B. abortus* replication in the placenta, we performed a mixed infection (1:1 ratio of *B. abortus* WT and *gluP* mutant) of pregnant mice bred to be conditionally deficient for proliferator-activated receptor γ (PPAR-γ) in macrophages (*Pparg*^fl/fl^ LysMcre^±^), as well as control mice, using the experimental design shown in Fig. 1A. At necropsy, we determined the competitive index or the ratio of WT to *gluP* mutant bacteria in tissues. As we have shown previously for non-pregnant mice (21), in splenic tissue during the acute phase of infection (13 days), the growth advantage of the WT strain over the *gluP* mutant in splenic tissue was not pronounced in either mouse strain (Fig. 2A). This result was expected, since in non-pregnant mice *B. abortus* does not utilize glucose to persist within splenic M2 macrophages until the chronic (60 days) phase of infection. In contrast, in the placentae of both PPAR-γ conditionally deficient mice and controls, *B. abortus* WT exhibited a strong (approximately 100-fold) competitive advantage over the *gluP* mutant that was not significantly different between mouse genotypes (Fig. 2B). These results suggest that placental M2-like macrophages are not the main site for glucose-dependent *B. abortus* replication in pregnant mice. However, fetal viability in the mice conditionally deficient for PPAR-γ in macrophages, increased, suggesting that M2-like macrophages in the placenta may play a role in *Brucella*-induced fetal death that is independent of intracellular glucose (Fig. 2C).

We next investigated whether glucose-dependent replication occurs in trophoblasts. To this end, we utilized the human BeWo choriocarcinoma line as an *in vitro* model for placental trophoblasts, as they model the replication of *B. abortus* in the endoplasmic reticulum that is observed in the ruminant placenta (23, 24). BeWo cells were inoculated with either *B. abortus* WT or the *gluP* mutant, and intracellular replication was monitored over 48 h using a gentamicin protection assay. In BeWo cells, *B. abortus* replicated between 4 and 48 h, and we observed that the *gluP* mutation had no effect on growth in these cells (Fig. 2D). These results suggest that trophoblasts are not likely to be the main placental niche in which glucose is utilized by *B. abortus* during placental infection. Taken together, these results demonstrate that neither PPAR-γ-expressing alternatively activated macrophages nor trophoblasts constitute the main niche for glucose-dependent *B. abortus* replication during placental infection.

### Glucose utilization by *B. abortus* partially contributes to placental pathology induced by infection

**Fig 3:**
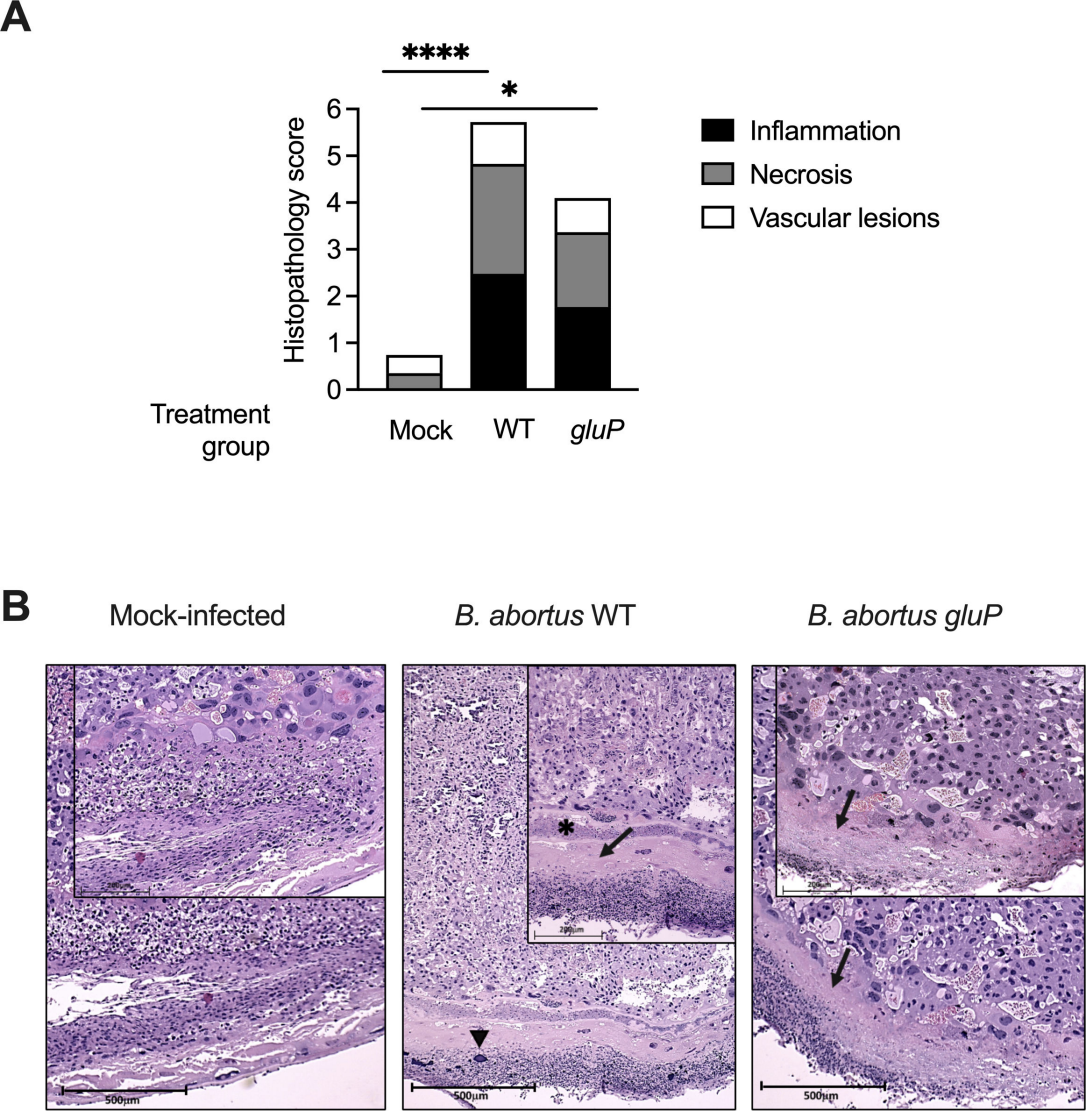
Glucose utilization contributes to *Brucella*-induced severe inflammation in the placenta. (**A**) Blinded histopathology scoring of placental tissue from mock-infected (*n* = 6), WT-infected (*n* = 7), or *gluP* mutant-infected mice (*n* = 5). Stacked bars show the median score for each criterion. Significance of differences between groups was analyzed using a Kruskal-Wallis test with Dunn’s post hoc test. *P < 0.05, ****P < 0.0001. (**B**) Representative images of hematoxylin and eosin-stained placental tissue from pregnant mice. The black arrows show necrotic areas; the triangle shows mineralization; and the asterisk shows nuclear debris infiltration (10×). The scale bar represents 500 µM. Images were obtained using a Zeiss Primo Star microscope with the brightness adjusted (Adobe Photoshop CS2).

Our results above (Fig. 1F) showed that glucose uptake contributed to fetal loss in *B. abortus-*infected pregnant mice. Since several studies (9, 11, 25) have linked placental inflammatory responses to *Brucella* infection with fetal death, we determined whether glucose transport played a role in placental inflammation during *B. abortus* infection by comparing histopathological lesions *in situ* in placentas from uninfected control mice and from mice infected individually with *B. abortus* WT or the *gluP* mutant. Placentas from mice infected with WT *B. abortus* exhibited evidence of moderate or severe histological lesions (histology score of 4–6), including an increased abundance of dead trophoblasts, increased neutrophil influx, and large necrotic areas, assessed by histopathology scoring in a blinded manner. In contrast, placental histological lesions were slight to absent in non-infected mice (histology score of 0–1). Placentas from mice infected with the *gluP* mutant exhibited an intermediate phenotype (histology score of 4), suggesting that glucose uptake contributes but is not essential to the ability of *B. abortus* to trigger placental inflammation (Fig. 3A and B). To determine whether glucose-dependent growth of *B. abortus* contributed to fetal loss, we analyzed the viability of pups in the *B. abortus*-infected and non-infected control dams. Consistent with what we have observed previously (Fig. 1F), in dams infected with WT *B. abortus*, almost all pups were deceased; in contrast, viability was significantly higher in the mice infected with the *gluP* mutant. Taken together, these results indicate that fetal death and reduced fertility triggered by *Brucella abortus* infection depend in part on its ability to transport glucose.

### Glucose uptake is not required for induction of trophoblast cell death by *B. abortus*

**Fig 4:**
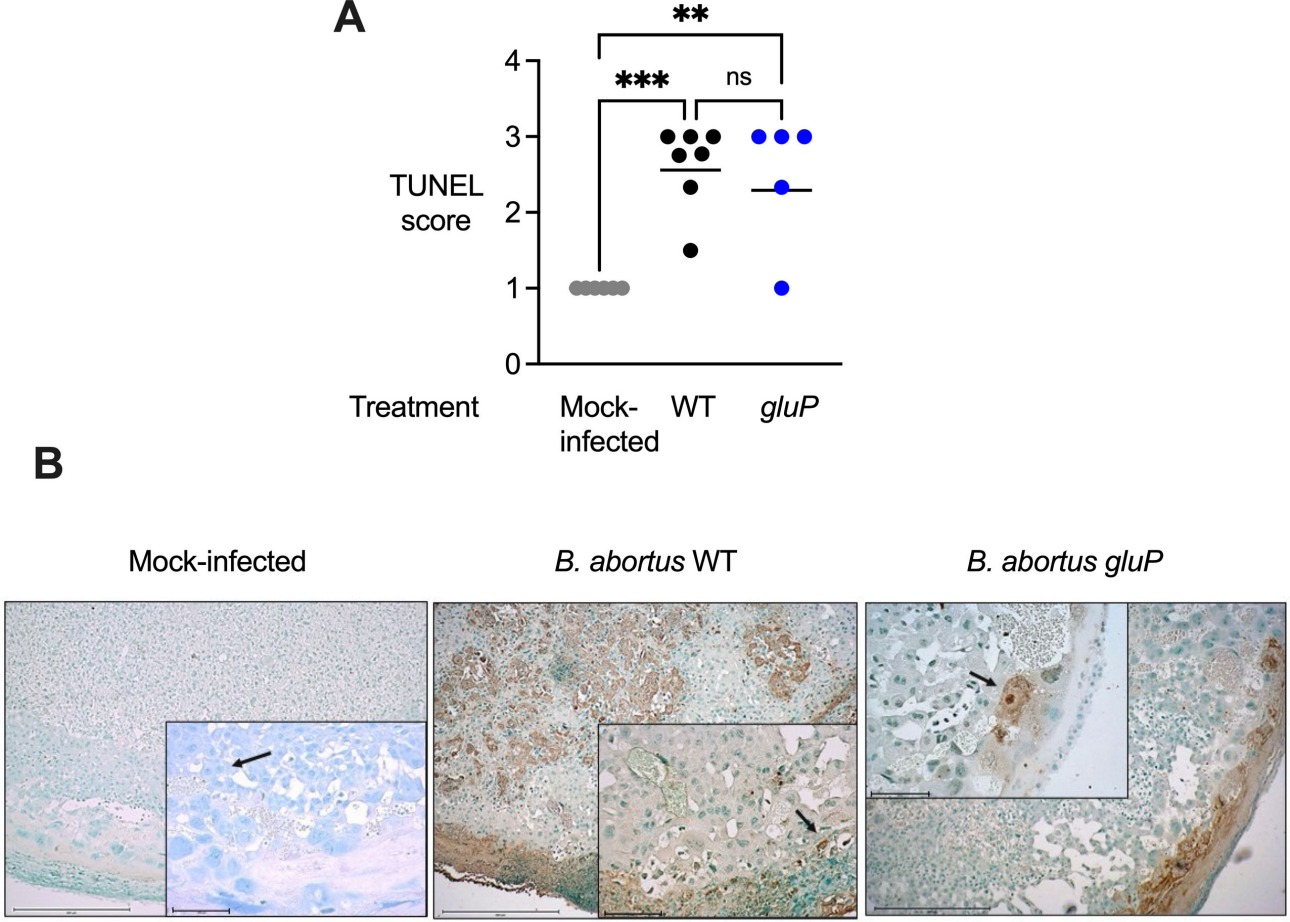
Impaired glucose transport in *B. abortus* does not affect trophoblast cell death in infected placenta. (A) Blinded histopathology scoring of TUNEL staining in the placental tissue of mock-infected, *B. abortus* WT-infected, or *gluP* mutant-infected mice. Values represent data for individual mice (gray, black, and blue circles) and means (black lines) (*n*  =  7) for the WT-infected group and *n*  = 5 for the GluP- and mock-treated groups. Two to four placentas were scored per mouse. **<0.01; ***P  <  0.005 using the Kruskal-Wallis test. (B) Representative images of TUNEL-stained placental tissue from non-infected, *B. abortus* WT-infected, or *gluP* mutant-infected pregnant mice. Black arrows point to trophoblasts that are positive for TUNEL staining in *B. abortus* WT- and *gluP*-infected placental tissue and TUNEL negative in non-infected placenta. Images were obtained using a Zeiss Primo Star microscope, and brightness was adjusted using Adobe Photoshop CS2.

Previous studies have established a correlation between trophoblast cell death and placental inflammation (9, 25). To gain a second line of evidence of death of trophoblasts in addition to the histological findings (Fig. 3), we detected the nuclear DNA fragmentation associated with cell death using terminal deoxynucleotidyl transferase dUTP nick-end labeling (TUNEL) staining, which identifies endonuclease-mediated DNA breakdown in the nuclei of apoptotic cells. We compared the abundance of apoptotic trophoblast cells *in situ* in placentas from three different groups of mice: non-infected controls, *B. abortus* WT-infected mice, and *gluP* mutant-infected mice. Although an expected low baseline level of trophoblastic cell apoptosis was observed in non-infected mice (Fig. 4A and B), in placentas from mice infected with *B. abortus* WT and *gluP* mutant, the analysis of TUNEL staining provided evidence for moderate to severe trophoblast death (Fig. 4A and B). Furthermore, we identified no difference in the number of TUNEL-positive trophoblasts between WT and *gluP* mutant-infected placentas, indicating that the trend for a lower inflammatory response in *gluP* mutant-infected placentas does not result from reduced cell death. Taken together with the glucose-independent replication of *B. abortus* in cultured BeWo cells (Fig. 2), these results suggest that *B. abortus* utilizes nutrients other than glucose to replicate within trophoblasts and trigger their apoptotic cell death.

## DISCUSSION

In this study, we show that glucose uptake contributes to the growth of *B. abortus* in the placental microenvironment of mice. *B. abortus* has been observed in the extracellular/interstitial space of the placenta and in the amniotic fluid of both ruminants and mice during placental infection (9, 12, 14, 26). At both of these sites, glucose is important for fueling the extracellular/interstitial growth of *B. abortus*. Limiting the extracellular growth of *B. abortus* in fetal tissues by abolishing its ability to acquire glucose attenuates its ability to elicit inflammation and cause fetal demise (Fig. 1C and 3). In contrast, in placental macrophages and trophoblasts, *B. abortus* appears to replicate independently of glucose. Reduction of available intracellular glucose by conditional deletion of *Pparg* in macrophages did not reduce placental growth of *B. abortus* (Fig. 2). However, a *B. abortus* mutant lacking the T4SS effector VceC, expressed during growth within macrophages, elicited less trophoblast cell death and rescued fetal viability (9, 10), suggesting that growth in both intracellular and extracellular placental niches contributes to the inflammatory pathology associated with fetal death (Fig. 2). These results add to our understanding of the nutritional environment encountered by *B. abortus* in the mouse placenta by pointing to distinct nutritional niches exploited by *B. abortus* during replication within placental macrophages and trophoblasts and in the extracellular/interstitial space, defined in part by different requirements for glucose uptake.

Studies of the metabolism supporting intracellular replication of *Brucella* spp. that characterizes acute and chronic, low-level infection of the mononuclear phagocyte system have revealed important insights into the nature of the nutritional niches exploited by *B. abortus* within phagocytic cells (17). These niches can change their characteristics between the acute and chronic stages of infection. During acute infection, phagocyte metabolism is shifted by proinflammatory cytokines, such as interferon-γ or by MyD88-dependent signaling pathways (27), to an M1-like profile, in which glycolytic metabolism provides energy for host cells. In contrast, during chronic infection, macrophages in tissue adopt a more M2-like physiology in which mitochondrial β-oxidation provides energy to the macrophage, and as a result, intracellular glucose becomes available to *B. abortus* (21). Consistent with GluP-mediated glucose uptake being required for maintenance of chronic *B. abortus* infection of the mononuclear phagocyte system (28), pyruvate kinase PykM was required to maintain chronic infection of the spleen, and gluconeogenic enzymes were required for chronic infection by *B. suis* biovar 5 (29), suggesting an important role for glucose utilization during chronic infection. In contrast to the intracellular utilization of glucose by *B. abortus* that was observed in the spleen in a chronic (60 days) infection model (21), our results suggest that the most important sites for glucose-fueled replication of *B. abortus* in the placenta are in the extracellular/interstitial space and amniotic fluid, rather than in placental M2 macrophages (Fig. 1 and 2).

Which metabolic pathways might enable *B. abortus* to utilize glucose to exploit the extracellular niche of the placenta? Since all *Brucella* spp. lack genes encoding phosphofructokinase, the first enzyme in the Embden-Meyerhof-Parnas glycolytic pathway, this pathway is unlikely to be used for glucose oxidation (16). The Entner-Doudoroff pathway is also defective in *B. abortus* 2308 due to an SNP in the *edd* gene encoding glucose-6-phosphate dehydratase, the first enzyme of this pathway, which results in a misfolded protein and suggests that this pathway would not be utilized for growth in the placenta (16). Therefore, the most likely pathway for glucose utilization in the placenta would be its oxidation to pyruvate via the pentose phosphate pathway, resulting in two NADPHs and one ATP. A scenario in which GluP-mediated uptake enables glucose oxidation to pyruvate via the pentose phosphate pathway in the placenta could explain the maintenance of this pathway in *Brucella*, despite it being dispensable for *B. abortus* growth in the mononuclear phagocyte system of mice (30).

Although classical studies (31) and newer research (16) have demonstrated that *Brucella* is capable of catabolizing glucose to pyruvate to feed the TCA cycle, glucose has consistently been dismissed as the preferred carbon source for *B. abortus*. In the ruminant placenta, erythritol has been suggested as the primary carbon source (13, 20). While a strong correlation between the presence of erythritol in fetal tissues of cows, sheep, and goats and the ability of *Brucella* spp. to utilize erythritol as a preferred carbon source is suggestive of a growth-promoting role in the ruminant placenta (19, 20), definitive (but difficult) studies to determine the requirements for glucose or erythritol as energy sources for *B. abortus* during placental growth in the natural bovine host using mutant strains incapable of glucose or erythritol utilization are still needed. In mouse tissues, Barbier et al. demonstrated, using a mutant lacking erythritol kinase (responsible for phosphorylating erythritol for use as an energy source), that erythritol was available but was not required for *B. abortus* multiplication in murine trophoblastic and macrophage-like cells, as well as in the mouse spleen and conceptus (fetus, placenta, and envelopes) (18). Our results suggest that while not the preferred carbon source for *B. abortus*, glucose may provide an alternative to erythritol utilization in the mouse placenta. Notably, previous studies have demonstrated that during pregnancy in mice, particularly in the process of decidualization, there is a marked increase in stromal glycogen levels in the placenta (32), suggesting its potential role in providing a readily mobilizable source of glucose to meet the high metabolic demands of the developing fetus. While the precise function of this glycogen reserve in the decidua remains unclear, it may serve as a crucial energy reservoir for *Brucella abortus* during placental infection of the mouse, supplying glucose that supports bacterial replication.

Our study has some limitations, including the anatomical differences in placentation between mice and cows, which may affect the placental environment encountered by *B. abortus* in both species. In addition, the physiology of the BeWo-transformed choriocarcinoma cell line used as a trophoblast model may differ from that of trophoblasts *in situ*, which may affect our conclusion that *B. abortus* growth within trophoblasts is independent of glucose uptake. Finally, we assayed bacterial colonization of the placenta at a late stage of infection, so our results do not rule out that glucose utilization within placental macrophages could be important in earlier stages of placental infection. Along these lines, it is possible that the inflammatory environment in the placenta drives macrophage metabolism toward glycolysis, rendering glucose unavailable to *B. abortus* for intracellular replication (21). This is, for example, the case in vaccinated hosts where development of specific antibodies drives polarization of macrophage metabolism in tissue toward glycolysis (33).

Taken together, our results suggest that the infected mouse placenta presents multiple nutritionally distinct niches for *B. abortus* replication and that in the extracellular/interstitial space, glucose uptake drives the massive replication that contributes to inflammation and fetal demise. These results add to our fundamental understanding of the environment encountered during *B. abortus* in the placenta, the site where in its natural bovine host its replication leads to abortion and transmission within the host population.

## MATERIALS AND METHODS

### Bacterial strains, media, and culture conditions

Bacterial strains used in this study were the virulent strain *B. abortus* 2308 and an isogenic mutant, BA159 (*gluP*), which has a miniTn*5* Km2 transposon insertion interrupting the *gluP* locus (34). The culture media used for *B. abortus* were tryptic soy agar (TSA; Difco/Becton, Dickinson, Sparks, MD), tryptic soy broth, or TSA plus 5% blood for bacterial inocula for mouse infection. For the culture of BA159, kanamycin (Km) was added to the culture medium at 100 µg/mL.

### Animal experiments

A murine placental infection model was utilized based on previous research (35). C57BL/6J mice aged 8–10 weeks were used. Mice were held in microisolator cages with sterile bedding and irradiated feed in a biosafety level 3 laboratory. Female C57BL/6J mice were mated with male C57BL/6J mice, and pregnancy was confirmed by the presence of a vaginal plug. At 5 days of gestation, pregnant mice were mock infected or infected i.p. with 10^5^ CFU of *B. abortus* 2308 or its isogenic *gluP* mutant. At 13 days after infection (18 days of pregnancy), the mice were euthanized by CO_2_ asphyxiation, and the spleen and placenta were collected aseptically at necropsy. At day 13 post-infection, fetal viability was evaluated based on the presence of fetal movement and heartbeat and on fetal size and skin color, and percent viability was calculated using the following formula: (number viable fetuses per litter / total number fetuses per litter) × 100. To collect amniotic fluid samples, the uterine horns were separated from the cervix and placed in a sterile petri dish. A sterile syringe with a 26 G needle was utilized to obtain amniotic fluid from amniotic sacs (amniotic fluid was clear, which indicates no contamination with embryo blood). Due to the small volume of amniotic fluid often obtained from each amniotic sac (~50 µL), amniotic fluid was obtained from two adjoining amniotic sacs and pooled. The number of viable bacteria in the amniotic fluid was determined by performing serial 10-fold dilutions in sterile phosphate-buffered saline (PBS) and plating on TSA. Then placenta and spleen samples were collected for bacteriological, histopathological, and TUNEL analyses.

Placentas and spleens from pregnant mice infected with *B. abortus* strains were collected in a 5 mL tube containing 1 mL of sterile PBS, and the tissue was homogenized using two scoops (1.2 g) of 0.9–2.0 mm stainless steel beads (Next Advance) and three satellite 3.2 mm stainless steel beads per tube in a Next Advance Bullet Blender 5 Storm homogenizer at Speed 10 for 5 min. The total tissue load of viable *B. abortus* was enumerated by performing serial 10-fold dilutions in sterile PBS and plating on TSA.

To approximate the proportion of intracellular and extracellular *B. abortus* spp. in tissue, 100 μL of the initial tissue homogenate was transferred to 900 μL of sterile solution containing 50 mg/mL of gentamicin (Invitrogen, Grand Island, NY). The samples were incubated for 30 min, followed by performing serial 10-fold dilutions in sterile PBS and plating on TSA. The proportion of gentamicin-sensitive (extracellular) bacteria in the tissue was approximated as follows: (total CFU before gentamicin treatment – gentamicin-resistant CFU) / total CFU. To control for potential differences in susceptibility of *B. abortus* WT and *gluP* mutant to gentamicin, we incubated 1 × 10^6^ CFU of cultured *B. abortus* 2308 and *gluP* mutant in the absence of host cells for 30 min in LB medium containing 50 µg/mL of gentamicin and found that for both strains, treatment resulted in killing of 94% of the *gluP* mutant and 93% of WT bacteria.

Female and male C57BL/6 *Pparg*^fl/fl^*LysM*^cre/−^ (Mac-PPARγ KO) and littermate control mice (*Pparg*^fl/fl^*LysM*^−/−^) were generated at UC Davis by mating *Pparg*^fl/fl^ mice with *LysM*^cre/cre^ mice (The Jackson Laboratory, Bar Harbor). Pregnant mice were inoculated at 5 days of gestation i.p. with 0.2 mL of PBS containing 10^5^ CFU of a 1:1 mixture of *B. abortus* 2308 and *gluP* mutant as previously described (36). At 13 days after infection (18 days of pregnancy), mice were euthanized by CO_2_ asphyxiation, and fetal viability was evaluated as described above. Spleens and placentas were collected as described above, and serial dilutions of the homogenate were plated on TSA and/or TSA + Km for enumeration of CFU.

### Mammalian cell culture

Intracellular multiplication in the BeWo (American Type Culture Collection CCL-98) human trophoblast-like cell line was determined as described previously (23). Cells were seeded in 24-well microtiter plates at a concentration of 2.5 × 104 cells/well in 0.5 mL of Ham’s F-12K (Thermo Fisher) supplemented with 10% fetal bovine serum and incubated for 24 h at 37°C in 5% CO_2_. For inoculum preparation, *B. abortus* 2308 or *gluP* mutant was grown for 24 h and then diluted in F-12K medium supplemented, and about 2.5 × 10^6^ bacteria were added to each well, corresponding to a multiplicity of infection of 100. Microtiter plates were centrifuged at 210 × *g* for 5 min at room temperature to synchronize infection. Cells were incubated for 25 min at 37°C in 5% CO_2_, then washed twice with d-PBS and then incubated with 50 µg/mL gentamicin (Gibco) in Ham’s F12-K for 30 min to remove free bacteria. Then, the medium was replaced with gentamicin-free medium, and the zero-time-point sample was taken as described below. In order to determine bacterial survival, the medium was aspirated at indicated time points after infection, and the cells were lysed with 0.5 mL of 0.5% Tween 20, followed by rinsing of each well with 0.5 mL of PBS. Viable bacteria were quantified by serial dilution in sterile PBS and plating on TSA. All experiments were performed independently in duplicate at least four times, and the standard error for each time point was calculated.

### Histopathology

Tissue samples were fixed by immersion in buffered 10% formalin, processed for paraffin embedding, sectioned in a microtome (4 μm-thick sections), and stained with hematoxylin and eosin. Histopathology scoring was performed as previously described (35). In short, formalin-fixed spleen and placenta tissue sections were stained with hematoxylin and eosin, and a veterinary pathologist performed an evaluation in a blind manner using previously described criteria (35). Scoring of placental lesions included four parameters: inflammation (neutrophilic infiltration): 0 (absence), 1 (mild focal infiltrate), 2 (moderate focal or multifocal infiltrate), and 3 (severe multifocal infiltrate); necrosis: 0 (absence), 1 (mild necrosis), 2 (moderate necrosis), and 3 (severe multifocal or focally extensive necrosis); vascular changes: 0 (absent) and 1 (mild microthrombi), 2 (moderate microthrombi), and 3 (marked microthrombi); and bacterial colonies associated with the lesion: 0 (absent), 1 (mild), 2 (moderate), and 3 (severe). Therefore, the combined histopathology score for placental lesions included all of these parameters, with a total score ranging from 0 to 12.

### TUNEL assay

Trophoblast death in formalin-fixed sections of the placenta was determined by a TUNEL assay using the ApopTag peroxidase *in situ* apoptosis detection kit (Millipore, Billerica, MA) following the manufacturer’s protocol. A veterinary pathologist performed a blinded evaluation, and a cell death score from 0 to 3 was assigned according to the intensity and distribution of stained dead cells in the tissue (0, zero to slight staining; 1, mild staining; 2, moderate staining; and 3, marked staining). Representative images were obtained using a Zeiss Primo Star microscope with the brightness adjusted (Adobe Photoshop CS2).

### Statistical analysis

For tissue culture experiments, statistical differences were calculated using a non-parametric Kruskal-Wallis test, and a *P* value of <0.05 was considered significant. To determine statistical significance in animal experiments, an unpaired Student’s *t*-test was used on log-transformed data. A two-tailed *P* value of <0.05 was considered significant. To assess statistical significance in animal experiments, we employed either the Mann-Whitney *U* test for non-parametric comparisons between two independent groups or one-way analysis of variance with post hoc analysis for multiple group comparisons, based on the experimental design and data distribution. Statistical analyses were conducted using GraphPad Prism.

## SUPPLEMENTAL MATERIAL

Fig. S1 iai.00060-25-s0001.docx Structure of the murine maternal-fetal interface and sampling sites for collection of amniotic fluid and placental tissue shown in Fig. 1.

ASM does not own the copyrights to Supplemental Material that may be linked to, or accessed through, an article. The authors have granted ASM a non-exclusive, world-wide license to publish the Supplemental Material files. Please contact the corresponding author directly for reuse.
