# Supplementary material for: Glucose uptake is essential for Brucella abortus growth in the extracellular space of the murine placenta
Source: Infect Immun. 2025 Mar 12;93(4):e00060-25. doi: 10.1128/iai.00060-25 (PMC11977311; doi:10.1128/iai.00060-25)

**SUPPLEMENTARY INFORMATION:**

**Figure S1**: Figure S1: Structure of the murine maternal-fetal interface and sampling sites for collection of amniotic fluid and placental tissue shown in Fig. 1 Made with BioRender.


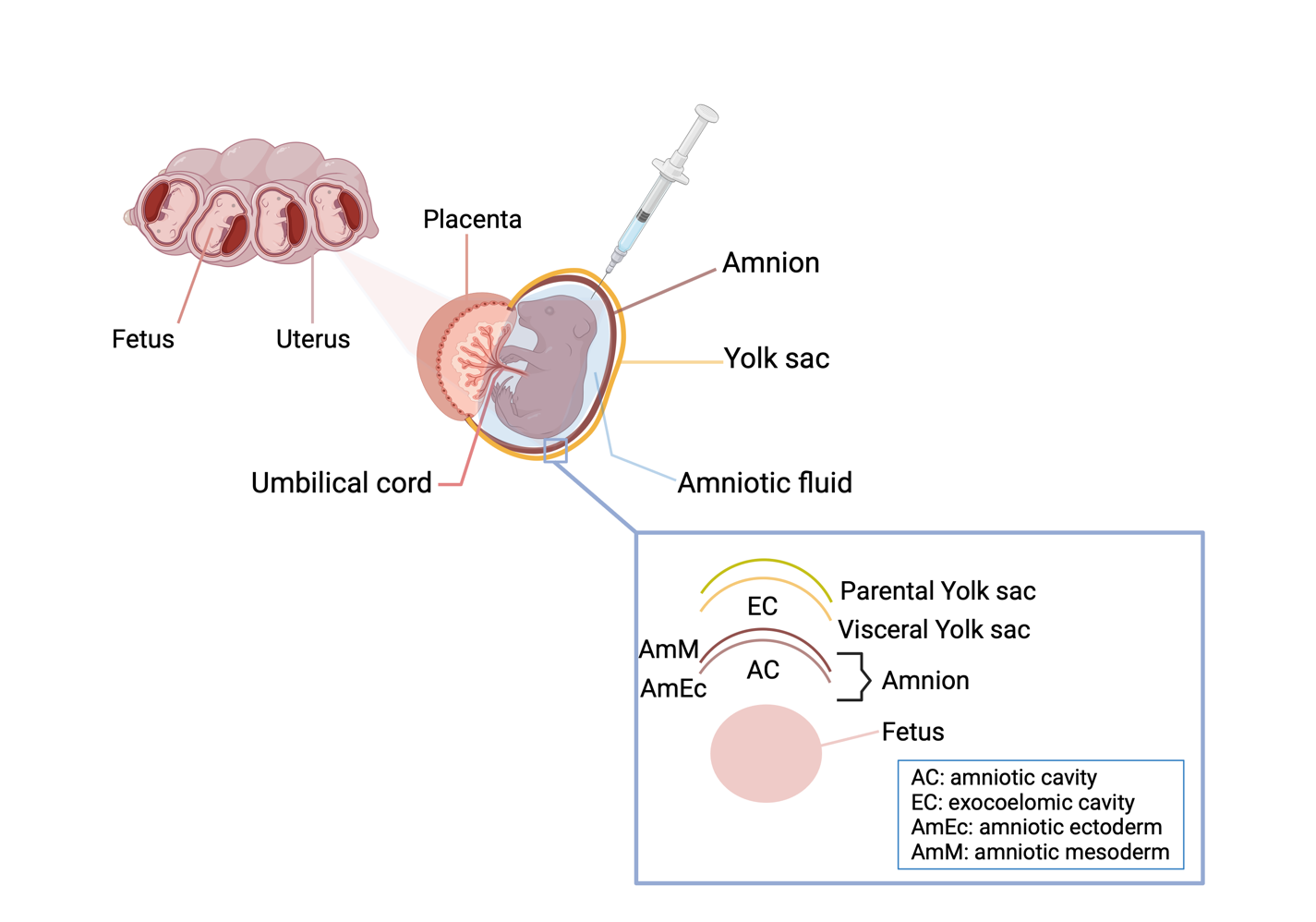

Supplement: Fig. S1 — Structure of the murine maternal-fetal interface and sampling sites for collection of amniotic fluid and placental tissue shown in Fig. 1. [file iai.00060-25-s0001.docx]
